# HPV Genotyping 9G Membrane Test: A Point-of-Care Diagnostic Platform

**DOI:** 10.3390/s141019162

**Published:** 2014-10-15

**Authors:** Keumsoo Song, Satish Balasaheb Nimse, Heejung An, Taisun Kim

**Affiliations:** 1 Biometrix Technology, Inc. 202 BioVenture Plaza, Chuncheon 200-161, Korea; E-Mail: hanlimsk@empal.com; 2 Institute for Applied Chemistry and Department of Chemistry, Hallym University, Chuncheon 200-702, Korea; E-Mail: satish_nimse@hallym.ac.kr; 3 Department of Pathology, College of Medicine, CHA University, Seongnam 487-801, Korea; E-Mail: hjahn@cha.ac.kr

**Keywords:** HPV, human papillomavirus, genotyping, cervical carcinoma, hybridization, 9G technology, cervical cytology

## Abstract

The results of HPV detection in 550 cervical samples by cervical cytology were compared with the sequencing analysis and HPV genotyping 9G membrane test. The HPV genotyping 9G membrane test can efficiently identify and discriminate five HR-HPV genotypes. The 100% identical results of HPV genotyping 9G membrane tests with the sequencing results in 550 clinical samples ensure its wide clinical applicability. The simple handling steps and the portable scanning device make the HPV genotyping 9G membrane test applicable in point-of-care settings. Moreover, the HPV genotyping 9G membrane test allows one to obtain final results in 30 min at 25 °C by simply loading the hybridization and washing solution and scanning the membranes without any drying steps or special handling. The clinical sensitivity and specificity of the HPV genotyping 9G membrane test was found to be 100%, which is much higher than cervical cytology.

## 1. Introduction

Cervical cancer is the second most common cancer of women in terms of both incidence and mortality worldwide [1,2]. Several studies indicate that persistent HPV infection with a high-risk type is strongly associated with cervical dysplasia [3] and carries a greater risk of the subsequent progression to cervical cancer [4]. The HPV testing assay, approved by the U.S. Food and Drug Administration (FDA) for primary screening, specifically tests for HPV types 16 and 18, the two high-risk types that contribute to roughly 70% of cervical cancers [5]. HPV also causes anal cancer, with about 85% of all cases caused by HPV16. HPV types 16 and 18 have also been found to cause 50% of vaginal, vulvar and penile cancers [6]. To overcome the limitations linked to cytologic cervical screening, HPV testing has been suggested for primary screening [7], triage of equivocal Pap smears or low-grade lesions [8] and follow-up after treatment for CIN [9]. Therefore, a highly proficient HPV genotyping test that can screen for the specific types of high-risk HPV for primary screening is essential.

The high mortality rate in HPV-infected patients necessitates the urgency to develop a rapid and field-deployable HPV genotyping test [10]. An HPV genotyping test must be capable of sensitive and specific HPV detection. Moreover, it must be simple to use and independent of complex laboratory instrumentation.

The giant magnetoimpedance (GMI)-based microchannel system for the quick and parallel genotyping of HPV16 and HPV18 has been published recently [11]. The GMI-based microchannel system allows efficient detection of HPV16 and HPV18 with copies of genomic DNA as low as five. The major disadvantage of this method is that it does not detect the other important HR-HPV genotypes, such as HPV31, HPV33 and HPV45. Moreover, though the dynamic range of detection of this assay is from five copies to 10^7^ copies, the assay fails to differentiate 200 to 10^5^ copies of the genomic DNA, which is critical in the follow up of the treatment.

Recently reported 9G DNAChip technology [12] demonstrates its unique applicability for genotyping of highly pathogenic viruses, such as human papillomavirus (HPV) [13] and human influenza virus (H1N1) [14]. The 9G DNAChips showed 100% clinically sensitive and specific genotyping results in clinical samples [15]. However, the dependence of the 9G DNAChips, like any other microarray platform, on costly instrumentation and highly-trained professionals limits its use in laboratory settings. Therefore, to address these problems, an HPV genotyping 9G membrane kit was developed for simple and convenient HPV genotyping [16]. The HPV genotyping 9G membrane kit allows one to obtain final results in 30 min with a very simple experimental protocol.

The clinical application of the HPV genotyping 9G membrane kit for the rapid detection and discrimination of HPV genotypes was evaluated by using 550 clinical samples. The HPV genotyping 9G membrane kit shows high sensitivity and specificity with 100% target-specific hybridization. The HPV genotyping 9G membrane kit allows the discrimination of genotypes with a ratio of 360:1 in 30 min at 25 °C using portable and inexpensive instrumentation. The HPV genotyping 9G membrane test can effectively genotype the five HR-HPV genotypes that are prevalent in cervical cancer cases. The HPV genotyping 9G membrane test demonstrates high clinical applicability indicated by the 100% agreement with the sequencing analysis. Out of 550 samples, 350 samples were found to be HPV positive. Out of 350 HPV-positive samples, 170, 60, 40, 39, 41 were found to be HPV16, HPV18, HPV45, HPV31 and HPV33, respectively. HPV16 and HPV18 together constituted almost 70% of all HPV-positive samples, HPV16 being highest at 49%.

## 2. Experimental Section

### 2.1. Materials

All chemicals were purchased from Sigma-Aldrich Chemicals, Seoul, Korea. All washing solvents for the substrates are of HPLC grade from SK Chemicals, Seoul, Korea. Ultrapure water (18 M·Ω/cm) was obtained from a Milli-Q purification system (Millipore, Darmstadt, Germany). Oligonucleotides were lined using a dispenser (BioDot Technologies, Inc., Irvine, CA, USA). Hybridization was done at 25 °C, and no special instrument was required. The fluorescence signal intensities were measured and analyzed by a BMT Membrane Reader™ (Biometrix Technology Inc., Chuncheon, Korea).

### 2.2. Composition of Different Solutions Used

(1) Immobilization solution (pH = 7.4): 15% glycerol, 50 mM butyl amine, 600 mM NH_4_Cl; (2) blocking buffer solution (pH = 7.4): 0.5% milk casein in 4 × SSC; (3) hybridization buffers (pH = 7.4): 25% formamide, 0.1% Triton X-100, 6 × SSC; (4) washing buffer solution (pH = 7.4): 0.1% SDS in 4 × SSC.

### 2.3. Typical Method for Preparation of the HPV Genotyping 9G Membranes

The HPV genotyping 9G membranes were obtained by a previously reported method [17,18]. The method for the immobilization of probes is briefly explained here. By lining the 18 pmol/μL solution of the oligonucleotide Probe1–Probe8 appended with 9 consecutive guanines (Table 1), the oligonucleotides can be immobilized on the AMCA membrane in 4 h.

After immobilization of the probes, as depicted in the Scheme 2, the membranes were soaked in the blocking solution and then dried to generate the HPV genotyping 9G membrane. Besides the probes complementary to the HPV genotypes, the probes (Probe6–Probes8) corresponding to the hybridization control (HC), PCR control (PCR,) and positive control (PC) were also lined on the membranes.

### 2.4. Clinical Samples

The samples were collected consecutively from 550 Korean women who visited the Department of Obstetrics and Gynecology, CHA Clinic, Bundang, Korea. The samples were collected by scraping the uterine cervical canal with a small cytobrush after Pap smear, and the brush was put into a 15 mL centrifuge tube containing phosphate-buffered saline. The specimens were collected as part of an informed consent protocol approved by the clinical studies committee of CHA hospital, Bundang, Korea.

### 2.5. Study Subjects

**Figure 1.**
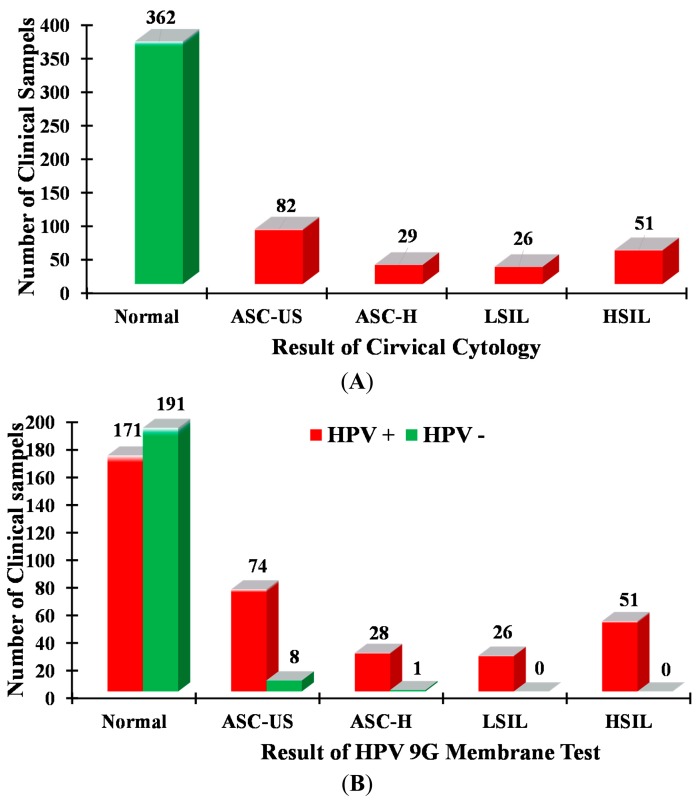
Identification of HPV infected samples. (**A**) Results of cervical cytology; (**B**) results of sequencing analysis and the HPV genotyping 9G membrane test (both results are in 100% agreement with each other).

The cervical cytology was performed on all 550 cervical samples. Out of 550 samples, HPV-positive results were found in the 188 samples, and the remaining 362 samples were found to be normal (no HPV detected) (Table 2). The 188 HPV-positive samples were categorized as ASC-US (atypical squamous cells of undetermined significance) (82, 43.6%), ASC-US-H (ASC-US, cannot exclude HSIL) (29, 15.4%), LSIL (low-grade squamous intraepithelial lesion) (26, 13.8%) and HSIL (high-grade squamous intraepithelial lesions) (51, 27.16%). The results of the cervical cytology were compared with the results of the sequencing analysis, as well as the HPV genotyping 9G membrane test results, as shown in Figure 1B and Table 3.

### 2.6. DNA Extraction and (PCR) Amplification

The MY09/MY11 primer set-mediated PCR (MY-PCR) and the GP5^+^/GP6^+^ primer set-mediated PCR (GP^+^-PCR) are the most frequently used amplification systems for the detection of HPV DNA in clinical samples, amplifying DNA fragments in the conserved L1 region with approximately 450 bp and 150 bp, respectively. Further, type-specific PCR primer sets allow the identification of individual genotypes. The MY11/GP6^+^ primer set consists of a fixed nucleotide sequence for the forward and reverse primers, respectively, and detects a wide range of HPV types by using a lowered annealing temperature during PCR [19,20].

The whole HPV genomic DNA extracted from clinical samples was amplified by duplex PCR to generate amplicons. HPV DNA was amplified with primers RP and FP (Table 1). The PCR mixture consisted of 10 μL of the extracted DNA, 10 μL of each primer (RP, FP), PCR premix (cat# K-2016V1, Bioneer Inc., Daejan, Korea) containing deoxyribonucleotide triphosphate, 2 U of Fast Start *Taq* DNA polymerase in an amplification buffer containing 2 mM MgCl2 and a tracking dye (Cy5). All tubes were incubated for 2 min at 50 °C before PCR was started. Amplification was performed with the following steps: pre-denaturation for 5 min at 94 °C, 45 cycles of 30 s each for denaturation at 94 °C; 45 cycles of 30 s each for annealing at 65 °C, 45 cycles of 30 s each for elongation at 72 °C and an final elongation step of 7 min at 72 °C. Five microliters of PCR product were subjected to agarose gel electrophoresis, using a 2% agarose standard run in 1X Tris borate EDTA. Ten microliters of this Cy5-labeled PCR product were used for further hybridization experiments in the HPV genotyping 9G membrane tests for HPV detection and genotyping. The whole procedure was followed in the case of the 550 clinical samples.

### 2.7. General Procedure for Hybridization, Washing, and Scanning

**Scheme 1.**
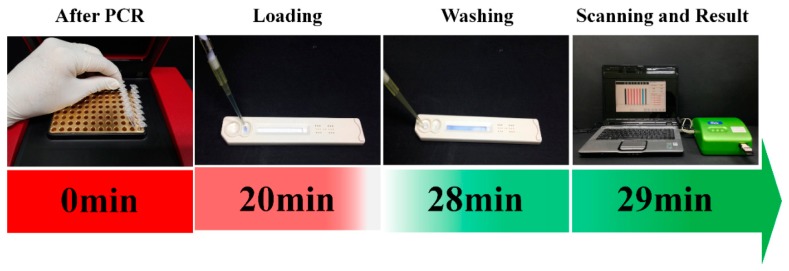
HPV genotyping 9G membrane, hybridization, washing, scanning and result.

The hybridization buffer was prepared by mixing 20 mL of hybridization solution and 600 μL of Cy5-HC-T1 (60 fmol/μL). A final 240 μL hybridization mixture was prepared by mixing the 220 μL of the hybridization buffer and 20 μL of the Cy5-labeled PCR product of the HPV genotype (e.g., HPV16, HPV18). Out of 240 μL, 110 μL of this hybridization mixture were loaded on the sample loading port on the HPV genotyping 9G membrane strip and allowed to hybridize for 20 min at 25 °C. After hybridization, washing solution was loaded into the washing port and allowed to stand for 8 min. Then, the HPV genotyping 9G membranes were scanned by the BMT Membrane Reader™ to obtain final results. Each experiment was done more than three times. The flow diagram for the hybridization, washing and scanning for the HPV genotyping 9G membrane test is depicted in the Scheme 1. The genotyping results can be obtained in 30 min by using HPV genotyping 9G membrane tests.

## 3. Results and Discussion

### 3.1. HPV Detection by Cervical Cytology

The samples collected consecutively from 550 Korean women were analyzed for the presence or absence of HPV infection by using cervical cytology. The obtained results are presented in Table 2 and Figure 1A. As shown in Figure 1A and Table 2, the results of cervical cytology demonstrated the presence of HPV infection in the 188 clinical samples with a distribution in ASC-US (82 cases), ASC-US-H (29 cases), LSIL (26 cases) and HSIL (51 cases). Moreover, the cervical cytology demonstrated the absence of HPV infection in 362 clinical samples.

It is very important to notice that although the cervical cytology indicated 362 normal samples out of 550 samples, the HPV genotyping 9G membrane test found that 171 samples were actually HPV positive, as demonstrated in Figure 1. Moreover, the cervical cytology detected false-positive results for about nine samples.

### 3.2. HPV Detection and Genotyping by the Sequencing

**Figure 2.**
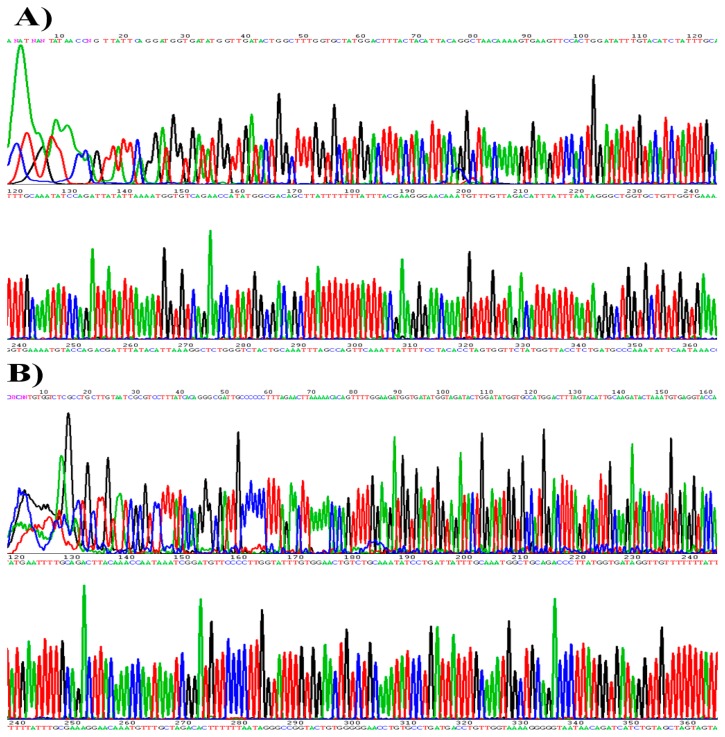
HPV genotyping by the sequencing analysis. (**A**) HPV16; (**B**) HPV18.

The primed PCR product was added to the sequencing reaction mixture. Sequencing was performed bi-directionally with the BigDye3 terminator cycle sequencing kit (PE Applied Biosystems) using the ABI PRISM 310 Genomic Analyzer (PE Applied Biosystems) at a dispensing pressure of 600 mbar with 8 ms open times and 65 s cycle times. The sequencing procedure was carried out by stepwise elongation of the primer strand upon cyclic dispensation of the different deoxynucleoside triphosphates (Amersham Pharmacia Biotech). A CCD camera detected the light output resulting from nucleotide incorporation. The data were obtained in a graphic format (Figure 2). Out of the 550 clinical samples, 350 samples were found to be HPV positive in the sequencing analysis, as demonstrated in Tables 2 and 3.

### 3.3. HPV Genotyping 9G Membrane Test

**Scheme 2.**
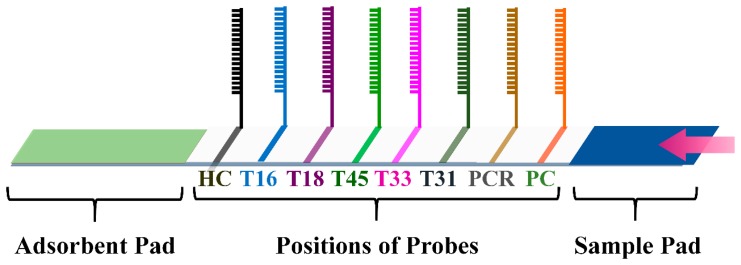
Positions of the immobilized probes on the membrane.

The HPV genotyping 9G membranes consist of the five HR-HPV type specific probes (HPV16, HPV18, HPV45, HPV31 and HPV33), one probe each for the positive control (PC), PCR and the hybridization control (HC), as shown in the Scheme 2.

The 5 μL of PCR product were subjected to agarose gel electrophoresis, and the product size of HPV DNA was found to be 250 base pairs (bp). For the detection and discrimination of the HPV genotypes in the clinical samples, 110 μL of hybridization mixture containing the Cy5-labeled PCR product of the HPV genotype (e.g., HPV16, HPV18 *etc*.) was loaded on the HPV genotyping 9G membrane test. The immobilized probes were allowed to hybridize with the Cy5-labeled PCR product of the HPV genotype for 20 min. Then, the washing solution was loaded into the washing solution loading port and allowed to stand for 8 min. After the hybridization and washing, the membranes were scanned to identify the HPV genotypes present in the clinical samples. Final results of the HPV genotyping 9G membrane test for 550 clinical samples are summarized in Table 3. Out of 550 samples, 350 samples were found to be HPV positive and 200 samples were found to be HPV negative.

As demonstrated in Table 3, the results of the HPV genotyping by the HPV genotyping 9G membrane tests were compared with those of the sequencing analysis and cervical cytology in the 550 clinical samples. Out of the 550 clinical samples, 350 samples (63.6%) were found to be HPV positive, and 200 samples were found to be HPV negative in the sequencing analysis. These results were 100% identical with that of the HPV genotyping 9G membrane tests, as shown in Tables 2 and 3.

**Figure 3.**
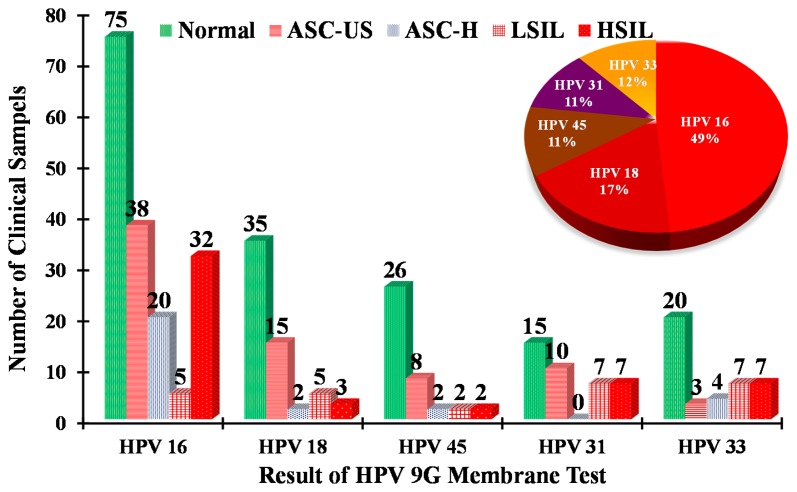
Distribution of HR-HPV genotypes in the samples categorized as normal, ASC-US, ASC-H, HSIL and LSIL by cervical cytology. The percentage distribution of five HR-HPV genotypes in the 350 clinical samples is depicted in the inset.

Out of 350 HPV-positive cases, 170, 60, 40, 39 and 41 were found to be HPV16, HPV18, HPV45, HPV31 and HPV33, respectively. As demonstrated in Figure 3 (inset), HPV16 accounts for 49% of cases, with 17%, 11%, 11% and 12% of cases being HPV18, HPV45, HPV31 and HPV33, respectively.

Overall, HPV16 and HPV18 altogether constituted almost 70% of all HPV-positive samples. According to the literature, HPV16 and HPV18 are responsible for 70% of all cervical cancer cases [21,22]. The results of the HPV genotyping 9G membrane test are in accordance with the data published in the literature.

The comprehensive analysis of the results in Figure 3 and Table 2 provides highly significant clinical information in the detection of the HR-HPV genotypes. The clinical significance of the HPV genotypes in normal, ASC-US, ASC-H, LSIL and HSIL is depicted in Figure 3. It is well known that almost 87% of cases of cervical cancers involve infection with the five HR-HPV types (HPV16, 18, 45, 31 and 33). The results of the present study Figures 1B and 3 demonstrate that the percentage of the five HR-HPV types (HPV16, 18, 45, 31 and 33) is 48.9% in the normal samples. This result indicates that the performance of cervical cytology may lead to false-negative results. Moreover, the percentage of the five HR-HPV types is 21.1% in the ASC-US stage, which is considered to be the early stage of HPV infection. It is very important to note that the HPV genotyping 9G membrane test detected 170 cases of HPV16. As demonstrated in Figure 3, 44.1% of the HPV16 cases were found in the samples considered as normal in cervical cytology. Moreover, 18.9% of the HPV16 positive cases were observed for HSIL, which is considered the beginning of the cancer stage. It is very important to note that the 38, 20, 5 and 32 HPV16 positive cases were found in ASC-US, ASC-H, LSIL and HSIL, respectively. The pattern of this high percentage of the HPV16 and HPV18 in the 550 clinical samples demonstrates the importance of the HPV genotyping 9G membrane tests. Therefore, the HPV genotyping membrane plays a vital role in clinical diagnosis of HPV infection, as it can detect and discriminate the five most important HR-HPV genotypes.

### 3.4. Limit of Detection

To determine the sensitivity of the HPV 9G membrane test in the detection and discrimination of five HR-HPV types, the hybridization solutions containing PCR products of the 1, 10, 10^2^, 10^3^ and 10^5^ copies of the respective HPV types were used for hybridization.

**Figure 4.**
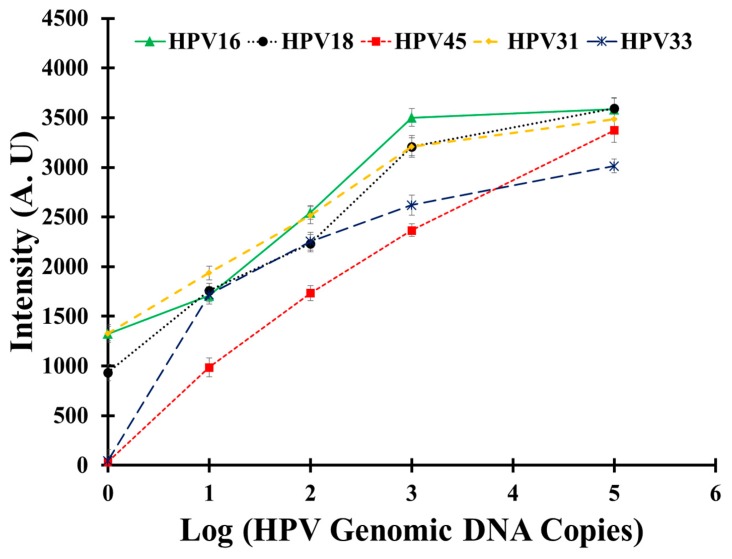
Standard curve of the fluorescent intensity corresponding to the number of copies of HPV DNA detected by using the HPV 9G membrane test.

As depicted in Figure 4, the obtained results clearly indicate that the HPV 9G membrane test can detect HPV16, HPV18 and HPV31 with as few as one copy of the respective HPV genomic DNA. Moreover, HPV33 and HPV45 can be efficiently detected with as few as 10 copies of the respective HPV genomic DNA. The HPV 9G membrane test demonstrates the comparative sensitivity with the reported methods in the detection of the Cy5-labeled PCR products [23]. Therefore, the HPV 9G membrane demonstrates high sensitivity in terms of the limit of detection. It is also very important to notice that the fluorescence signal increases with the increase in the number of copies of genomic DNA.

### 3.5. Statistical Analysis

The accuracy of the HPV genotyping 9G membrane tests for the detection of the HPV genotypes in the 550 clinical samples was calculated from the sensitivity (true-positive rate) and specificity (true-negative rate) of 95% confidence intervals (CI). To determine clinical sensitivity and specificity, the positive samples, both by sequencing analysis and by HPV genotyping 9G membrane tests, were defined as true positive. The negative samples, both by sequencing analysis and by HPV genotyping 9G membrane tests, were defined as true negative.

### 3.6. Discussion

Recently, we have reported the HPV genotyping 9G membrane and HPV2 genotyping 9G membranes used for the genotyping of the five HR-HPV genotypes and the screening of the 14 HR-HPV genotypes, respectively. Moreover, for the PCR line in the HPV2 genotyping 9G membranes was used for the detection of the presence of the low-risk HPV genotypes (LR-HPV) along with the 14 HR-HPV genotypes [17]. However, in the present article, the HPV genotyping 9G membrane test is designed for the genotyping of the five HR-HPV (HPV16, HPV18, HPV45, HPV31 and HPV33) genotypes, which are prevalent in almost 87% of cervical cancer cases. The HPV genotyping 9G membrane was designed in such a way that the probes immobilized on the five lines were used for the highly-specific detection of the five HR-HPV (HPV16, HPV18, HPV45, HPV31 and HPV33) genotypes. Moreover, the probe immobilized on the PCR line was designed for the screening of the 14 other HR-HPV genotypes.

The HPV 9G DNA chip test reported earlier [15] was designed for the detection and discrimination of 14 HR-HPV genotypes and five LR-HPV genotypes in the clinical samples. The HPV 9G DNAChip test allows one to detect single and multiple infections in 40 min at 25 °C. Moreover, the HPV 9G DNAChip test showed 100% clinical sensitivity and specificity. However, the requirement of costly instrumentation is a major hurdle for the application of the HPV 9G DNAChip test in the laboratory setting.

An HPV genotyping 9G membrane test is presented in this article, which addresses the problems faced by the HPV 9G DNAChip test. The HPV genotyping 9G membrane tests take only 30 min for the highly specific detection of HPV genotypes. As shown in Scheme 1, the HPV genotyping 9G membrane uses simple hybridization and washing steps, which allows it to be used in small laboratories and does not need highly-trained professionals. Furthermore, the use of a very small portable scanner makes the HPV genotyping 9G membrane tests suitable for point-of-care settings.

One hundred percent clinical sensitivity, as well as 100% clinical specificity were observed for HPV genotyping by the HPV genotyping 9G membrane tests. The clinical sensitivity and specificity of cervical cytology was 67.2% and 95.7%, respectively. The positive predictive value and negative predictive value of cervical cytology were 97.5% and 53.9%, respectively. On the contrary, the HPV genotyping 9G membrane test demonstrated 100% clinical sensitivity, clinical specificity, positive predictive value and negative predictive value.

The results of the HPV genotyping 9G membrane test detected that many clinical samples (362) designated as normal in cervical cytology were found to be high-risk-type HPV genotypes (171, 48.9%). The low sensitivity and specificity of cervical cytology may lead to inappropriate judgment. On the contrary, the HPV genotyping 9G membrane test showed accuracy in the genotyping of HPV types in the clinical samples.

The major disadvantages of the commercial DNA chips are that they require complicated steps and highly-trained professionals. It is important to notice that, in the case of the HPV genotyping 9G membrane test, the final results can be obtained very easily. Because, the hybridization solution and the washing solution are directly loaded on the membranes, the final results can be obtained by simply scanning the membranes. These simple steps make the test an appropriate platform for accurate HPV genotyping. The HPV genotyping 9G membrane test is highly accurate for the detection and discrimination of HR-HPV genotypes in the clinical diagnosis indicated by the 100% sensitivity and specificity.

The results for the sequencing of the HPV genotypes, HPV16 (49%), HPV18 (17%), HPV45 (11%), HPV31 (11%) and HPV33 (12%), were 100% identical with the HPV genotyping 9G membrane test. The 63.8% (18.8% HPV16, 5% HPV16, 5% HPV45, 17.9% HPV31 and 17.1% HPV33) of HR-HPV genotypes that were found to cause HSIL were detected by the HPV genotyping 9G membrane test. Similarly, the 51.3% (2.9% HPV16, 8.3% HPV18, 5.0% HPV45, 17.9% HPV31, and 17.1% HPV33) of HR-HPV genotypes that were found to cause LSIL were detected by the HPV genotyping 9G membrane test.

The results of cervical cytology demonstrate that, out of 550 samples, 362, 82, 29, 26 and 51 samples were normal, ASC-US, ASC-H, LSIL and HSIL, respectively (Table 2). It is interesting to notice that the present test demonstrates that 171 samples out of 362 normal samples in cervical cytology were actually HPV positive. The ability of the HPV genotyping 9G membrane test to detect HPV genotypes in clinical samples is credited to its sensitivity. The limit of detection of the HPV genotyping 9G membrane test for the detection of five HPV genotypes (HPV16, HPV18, HPV31, HPV33 and HPV45) is in the range of 10–100 copies [15].

The HPV genotyping 9G membrane test is an accurate method for the detection and genotyping of HPV in clinical samples. The genotyping results of the HPV genotyping 9G membrane tests were in 100% agreement with the results of the sequencing analysis. The HPV genotyping 9G membrane test on the 550 clinical samples proved that the preliminary findings of cervical cytology of HPV-infected samples may lead to the false detection of HPV.

PCR techniques, including TaqMan PCR, also represent a sensitive method for the detection of HPV DNA. In another method, the standard nested HPV PCR can be performed using type-specific primers followed by direct sequencing of the PCR product. However, these assays have demonstrated only 85%–90% clinical sensitivity and specificity, which are inadequate for direct clinical applications.

## 4. Conclusions

The HPV genotyping 9G membrane test makes the detection and discrimination of HPV genotypes in a clinical sample very easy by using a simple experimental protocol. The HPV genotyping 9G membrane test allows one to obtain final results in 30 min at 25 °C simply by loading the hybridization solution containing the PCR product and the washing solution on the membrane. The final results can be obtained by scanning the membranes without any drying steps or special handling. The simple handling steps make the present test a very convenient platform for accurate HPV genotyping.

The accuracy of the HPV genotyping 9G membrane test for HPV genotyping was assured by the 100% agreement with the results of sequencing analysis. The HPV genotyping 9G membrane tests demonstrate a clinical value in decision-making, as they can identify and discriminate the five HR-HPV genotypes. The efficient detection and discrimination of the 550 clinical samples make the HPV genotyping 9G membrane test a promising diagnostic tool for accurate HPV genotyping.

## Figures and Tables

**Table 1.** Sequences of the probes immobilized on the HPV genotyping 9G membranes.

**Line**	**Probes**	**Type**	**Sequence**
T16	Probe1	HPV16	5′-GGGGGGGGG AAA TAC AAA GTA CCT ACG ACA AGG GGA GG-3′
T18	Probe2	HPV18	5′-GGGGGGGGG AAA TAC AAA GTA TAG CAG ACT TGT TGA GG-3′
T45	Probe3	HPV45	5′-GGGGGGGGG AAA TAC AAA GTA TAG TAG ACA AGT GGA GG-3′
T33	Probe4	HPV33	5′-GGGGGGGGG AAA TAC AAA ATA TAT AAG ACA AGT TGA AG-3′
T31	Probe5	HPV31	5′-GGGGGGGGG AAA TAC AAA GTA TTT AAG ACA AGG TGA GG-3′
HC	Probe6	HC	5′-GGGGGGGGG CTT TAT TTT CC ACT GTT CTC GGC ACG-3′
PCR	Probe7	PCR	5′-GGGGGGGGG CTT TAT CTT GAC ATG KKG AGG AAT ATG A-3′
PC	Probe8	PC	5′-GGGGGGGGG TGA TTT ACA GTT TAT DTT TC-3′

Target1 (HC-Cy5-T1)		HC-Cy5	3′-GGATCACCGAGATACCATTGGAGACTGCG-Cy5-5′
Forward primer		FP	3′-GCMCAGGGWCATAAYAATGG-5′
Reverse primer		RP-Cy5	3′-GAAAHATAAACTGTAAATCATAYTC-Cy5-5′

HC, probe for the hybridization control; PC, probe for the primer control (positive control); PCR, probe for the PCR control; HC-Cy5-T1, Target oligonucleotide for the HC probe; GGGGGGGGG, 9G for immobilization of the probes on the AMCA slides; AAA TAC AAA, vertical spacer groups.

**Table 2.** Comparison of the results of HPV genotyping by cervical cytology with the results of the sequencing analysis and the HPV genotyping 9G membrane test (550 clinical samples).

**Cervical Cytology**		**Sequencing Analysis**		**HPV Genotyping 9G Membrane Test**	

		**HPV**+	**HPV**−	**HPV**+	**HPV**−
Normal	362 (65.8%)	171 (47.2%)	191	171 (47.2%)	191
ASC-US	82 (14.9%)	74 (90.2%)	8	74 (90.2%)	8
ASC-H	29 (5.3%)	28 (96.6%)	1	28 (96.6%)	1
LSIL	26 (4.7%)	26 (100%)	0	26 (100%)	0
HSIL	51 (9.3%)	51 (100%)	0	51 (100%)	0

ASC-US, atypical squamous cells of undetermined significance; ASC-H, atypical squamous cells cannot exclude HSIL; LSIL, low-grade squamous intraepithelial lesion; HSIL, high-grade squamous intraepithelial lesions.

**Table 3.** Comparison of the results of HPV genotyping by the sequencing analysis and HPV genotyping 9G membrane test in the 550 clinical samples.

**HPV Type**	**Normal**		**ASC-US**		**ASC-H**		**LSIL**		**HSIL**	
				
	**A**	**B**	**A**	**B**	**A**	**B**	**A**	**B**	**A**	**B**
HPV16	75	75	38	38	20	20	5	5	32	32
HPV18	35	35	15	15	2	2	5	5	3	3
HPV45	26	26	8	8	2	2	2	2	2	2
HPV31	15	15	10	10			7	7	7	7
HPV33	20	20	3	3	4	4	7	7	7	7
Negative	191	191	8	8	1	1				

A, results of sequencing analysis; B, results of the HPV genotyping 9G membrane test.
